# Validation of a noninvasive aMMP‐8 point‐of‐care diagnostic methodology in COVID‐19 patients with periodontal disease

**DOI:** 10.1002/cre2.589

**Published:** 2022-07-11

**Authors:** Shipra Gupta, Ritin Mohindra, Mohita Singla, Sagar Khera, Amit Kumar, Nilminie Rathnayake, Timo Sorsa, Andreas Pfützner, Ismo T. Räisänen, Roop K. Soni, Poonam Kanta, Akanksha Jain, Krishan Gauba, Kapil Goyal, Mini P. Singh, Arnab Ghosh, Kamal Kajal, Varun Mahajan, Vikas Suri, Ashish Bhalla

**Affiliations:** ^1^ Unit of Periodontics, Oral Health Sciences Centre Post Graduate Institute of Medical Education and Research (PGIMER) Chandigarh India; ^2^ Department of Internal Medicine Post Graduate Institute of Medical Education & Research (PGIMER) Chandigarh India; ^3^ Department of Oral and Maxillofacial Diseases University of Helsinki and Helsinki University Hospital Helsinki Finland; ^4^ Department of Dental Medicine, Division of Periodontology Karolinska Institutet Huddinge Sweden; ^5^ Clinical Research Department, Diabetes Center and Practice Pfützner Science and Health Institute Mainz Germany; ^6^ Department of Virology Post Graduate Institute of Medical Education and Research (PGIMER) Chandigarh India; ^7^ Department of Anaesthesia and Intensive Care Post Graduate Institute of Medical Education and Research (PGIMER) Chandigarh India

**Keywords:** biomarkers, oral health, periodontitis, SARS CoV‐2

## Abstract

**Objectives:**

The aim of this study was to validate an active matrix metalloproteinase (MMP‐8) point‐of‐care diagnostic tool in COVID‐19 patients with periodontal disease.

**Subjects, Materials, and Methods:**

Seventy‐two COVID‐19‐positive and 30 COVID‐19‐negative subjects were enrolled in the study. Demographic data were recorded, periodontal examination carried out, and chairside tests run for evaluating the expression of active MMP‐8 (aMMP‐8) in the site with maximum periodontal breakdown via gingival crevicular fluid sampling as well as via a mouth rinse‐based kit for general disease activity. In COVID‐19‐positive patients, the kits were run again once the patients turned COVID‐19 negative.

**Results:**

The overall (*n* = 102) sensitivity/specificity of the mouthrinse‐based kits to detect periodontal disease was 79.41%/36.76% and that of site‐specific kits was 64.71%/55.88% while adjusting for age, gender, and smoking status increased the sensitivity and specificity (82.35%/76.47% and 73.53%/88.24, respectively). Receiver operating characteristic (ROC) analysis for the adjusted model revealed very good area under the ROC curve 0.746–0.869 (*p* < .001) and 0.740–0.872 (*p* < .001) (the aMMP‐8 mouth rinse and site‐specific kits, respectively). No statistically significant difference was observed in the distribution of results of aMMP‐8 mouth rinse test (*p* = .302) and aMMP‐8 site‐specific test (*p* = .189) once the subjects recovered from COVID‐19.

**Conclusions:**

The findings of the present study support the aMMP‐8 point‐of‐care testing (PoCT) kits as screening tools for periodontitis in COVID‐19 patients. The overall screening accuracy can be further increased by utilizing adjunctively risk factors of periodontitis. The reported noninvasive, user‐friendly, and objective PoCT diagnostic methodology may provide a way of stratifying risk groups, deciding upon referrals, and in the institution of diligent oral hygiene regimens.

## 1 INTRODUCTION

Respiratory failure due to acute respiratory distress syndrome (ARDS) has been implicated to be the most prominent cause for COVID‐19‐related mortality (Malek et al., 2020). Severe acute respiratory syndrome coronavirus 2 (SARS CoV‐2) reportedly has the potential to induce pulmonary tissue alterations via numerous pathways, of which one involves matrix metalloproteinases (MMPs; Malek et al., 2020). This pathophysiology is an uncanny reflection of that involving SARS CoV‐2. MMPs lead to the degradation of extracellular matrix and modify the immune response. Besides mediating pulmonary tissue remodeling, these factors consequentially relate to increased vascular permeability along with endothelium damage (Malek et al., 2020). Mechanical ventilation for ARDS management can further cause lung injury as a result of ventilation‐induced MMP‐8 elaboration (Kong et al., 2020; Malek et al., 2020). In fact, the eventual mortality of patients has been associated with the elevated levels of MMP‐2, MMP‐9, MMP‐8, and TIMP‐1, as observed in the early stages of sepsis (Lauhio et al., 2011). MMP‐8 expression has also been evidenced to be indicative of systemic compromise and multiorgan pathoses (Lauhio et al., 2011). There is ample evidence to support the association between compromised clinical outcomes and elevated levels of certain systemic inflammatory biomarkers. Reportedly, MMPs also play a role in enhancing early viral entry into cells (Yates et al., 2020).

Periodontitis is one of the most prevalent chronic inflammatory oral diseases. Proteinases, pertinent of which, collagenase, responsible for bringing about matrix degradation in the presence of periodontitis is primarily derived from polymorphonuclear leukocytes (PMNs) of the diseased periodontium. Periodontopathogens of potency can elaborate proteases responsible for activating latent MMP‐1 and ‐8 by direct proteolysis while instigating their secretion from gingiva‐derived fibroblasts and oral keratinocytes, as well as via infiltrating inflammatory cells (Ding et al., 1996; Gupta, Sahni et al., 2021; Sorsa et al., 1995, 2006). Subsequent to PMN release, these latent MMPs transform into their activated counterparts as a result of proteolytic cleavage or reactive oxygen species interaction (Ding et al., 1996). Elevated levels of PMN‐derived collagenolytic MMP‐8 have been evidenced in the saliva, gingival crevicular fluid (GCF), and gingival tissue of periodontitis patients (Ding et al., 1997; Gangbar et al., 1990; Kiili et al., 2002; Lee et al., 1995; Mancini et al., 1999; Romanelli et al., 1999; Sorsa et al., 2015, 2006). The rapidity of PMN degranulation accompanied by the enzyme release upon microbial phagocytosis provides further strength to the validity of this pathway in the pathophysiology of periodontitis (Ding et al., 1997).

An active MMP‐8 (aMMP‐8) point‐of‐care (PoC) test, has been validated across countries in both the adolescent and adult populations as a tool to define active and inactive sites of periodontal disease, evaluate prognosis, and assess patients during the treatment and maintenance phases (Alassiri et al., 2018; Heikkinen et al., 2016; Izadi Borujeni et al., 2015; Johnson et al., 2016; Lähteenmäki et al., 2020; Leppilahti et al., 2018; Lorenz et al., 2017; Nwhator et al., 2014; Räisänen et al., 2018, 2019; Sorsa et al., 2020, Sorsa, Grigoriadis et al., 2021). This PoC testing methodology, in particular, exhibits a sensitivity of 76%–83% and specificity of 96% with results being reported in a time frame of 5–7 min (Sorsa et al., 2015, 2017).

Of late, numerous hypotheses and some studies have suggested the possibility of a link between periodontal disease and COVID‐19 adverse outcomes (Gupta & Sahni 2020; Gupta, Saarikko et al., 2022; Gupta, Mohindra et al., 2022; Gupta, Sorsa et al., 2022; Sahni & Gupta, 2020). In fact, SARS‐CoV‐2 has also been recovered from GCF, further strengthening its rationale to be utilized as a diagnostic fluid (Gupta, Mohindra et al., 2021). Identification of this potential corisk factor by means of a PoC tool would be invaluable to healthcare personnel and medical professionals in deciding referrals, identifying patient risk groups, and reinforcing oral hygiene measures (Räisänen et al., 2021; Sorsa, Sahni et al., 2021).

It could also mitigate and predict the risk of adverse systemic effects of periodontitis and potentially also the risk of severe COVID‐19 infections, especially among older patients and those with underlying health conditions. Moreover, it could also increase awareness of the periodontal disease among individuals and encourage and warn them to seek oral care before periodontal disease has progressed to a severe stage. aMMP‐8 could then be used as a potential biomarker for COVID‐19 infection and help in understanding the inflammatory response against the virus.

The current study thus aimed to assess whether such a PoC diagnostic methodology could be utilized as a screening tool to assess active periodontal disease in patients suffering from COVID‐19.

## 2 MATERIALS AND METHODS

The cross‐sectional analytical study was carried out by the Unit of Periodontics, Oral Health Sciences Centre, Postgraduate Institute of Medical Education and Research (PGIMER), Chandigarh, India. Due approval was taken from the Institute Ethics Committee (INT/IEC/2021/SPL‐453 & 636). The present study conforms to STROBE guidelines.

One hundred and two COVID‐19 suspected patients reporting to PGIMER, Chandigarh were enrolled in the study between January 15, 2021 and February 20, 2021. Their COVID‐19 status was evaluated at the Communicable Disease ward of the Department of Virology, by nasopharyngeal swab testing. Seventy‐two turned out to be positive for COVID‐19, while 30 were COVID‐19 negative. COVID‐19 positive patients were then either quarantined at home or admitted to the hospital. The patient information sheet was given to all the patients and written informed consent was obtained from all the subjects. Pregnant ladies, patients less than 18 years old, and those unwilling or not in a position to give written informed consent were excluded from the study. The sample size was based on convenient sampling as close proximity to a potentially infectious person is required to conduct intraoral examination and aMMP‐8 analysis. Demographic data were recorded, and chairside tests were run for evaluating the expression of aMMP‐8 in the site with the maximum periodontal breakdown as well as via a mouth rinse‐based kit for general disease activity. In the COVID‐19 positive patients, the kits were run again once the patients recovered and tested COVID‐19 negative. Hence the subjects were recalled for the second examination at 17 days. The clinical examination was carried out by a single examiner (S. G.), while the kits were run by another examiner (M. S.).

### 2.1 Patient‐related characteristics

#### 2.1.1 Covariates

Patient characteristics like age, sex, smoking habits, and other COVID‐related comorbidities/risk factors such as diabetes, hypertension, pulmonary disease, chronic kidney disease, cancer, coronary artery disease, obesity, and any other comorbidity were recorded.

### 2.2 Periodontal clinical examination

Periodontal clinical examinations were conducted using a 10‐mm round‐tip manual Williams's periodontal probe. All permanent teeth, excluding the third molars, were examined at six sites per tooth (disto‐buccal, mid‐buccal, mesiobuccal, disto‐palatal, mid‐palatal, mesio‐palatal). Gingival recession, gingival marginal level, periodontal probing depth, bleeding on probing (BOP), number of teeth present/missing/carious were recorded. Clinical attachment loss was calculated. Patients were categorized into: periodontally healthy, gingivitis, and stage I–IV periodontitis, as per the new classification of periodontitis as described by Chapple et al. (2018), Trombelli et al. (2018), and Tonetti et al. (2018), based on their clinical examination alone, as conducting intraoral radiographs for COVID‐positive patients was not feasible (Chapple et al., 2018; Tonetti et al., 2018; Trombelli et al., 2018).

### 2.3 aMMP‐8 PoC mouth rinse sample collection and qualitative analysis

Mouthrinse sample collection was conducted step by step according to the manufacturer's instructions for PerioSafe lateral‐flow immunoassay kits (PerioSafe; Dentognostics GmbH, Solingen, Germany) utilizing two anti‐aMMP‐8 monoclonal antibodies as described previously (Hanemaaijer et al., 1997; Sorsa et al., 1999). Patients were made to rinse with tap water for 30 s and then spit it out. After 1 minute, they were asked to rinse their mouth for 30 s with the rinsing solution provided in the test kit and spit it out in the measuring cup provided in the kit. About 2–3 ml of sample solution was collected in the syringe provided in the kit and the filter attached. The test cassette was kept on a horizontal surface and 3–4 drops of the sample solution were poured into the round opening of the test cassette. The results were read after exactly 5 min.

### 2.4 aMMP‐8 PoC site‐specific sample collection and qualitative analysis

Site‐specific sample collection was conducted step by step according to the manufacturer's instructions for ImplantSafe lateral‐flow immunoassay kits (ImplantSafe; Dentognostics GmbH, Jena, Germany) utilizing, similar to the PerioSafe kit, two anti‐aMMP‐8 monoclonal antibodies as described previously (Hanemaaijer et al., 1997; Sorsa et al., 1999). The sampling site was prepared by the removal of excess saliva with a short, gentle blast of air/cotton swab. A sterile collection strip provided in the kit was placed apically as deeply as possible into the sulcus at the sampling site using tweezers, with its blue end pointing away from the tooth. The strip was left there for 30 s and then pulled out. It was placed in the vial containing elution fluid provided in the kit. The vial was gently turned upside down five times to make sure the strip was totally immersed in the fluid. Once the strip starts floating, it was tipped upside down five times again. The samples were analyzed by a dipstick, which was dipped with the yellow absorption zone facing down, into the elution fluid until the liquid was visible in the readout window. After that, the dipstick was removed from the elution fluid and placed on a level surface. The results were read after exactly 5 min.

**Figure 1:**
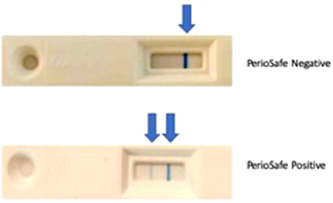
An aMMP‐8 PoC lateral‐flow immunoassay mouth rinse test. A negative (one blue line) test result indicates aMMP‐8 levels <20 ng/ml in mouth rinse; and a positive test result (two blue lines) aMMP‐8 levels of ≥20 ng/ml in mouth rinse. aMMP‐8, active matrix metalloproteinase 8; PoC, point‐of‐care.

**Figure 2:**
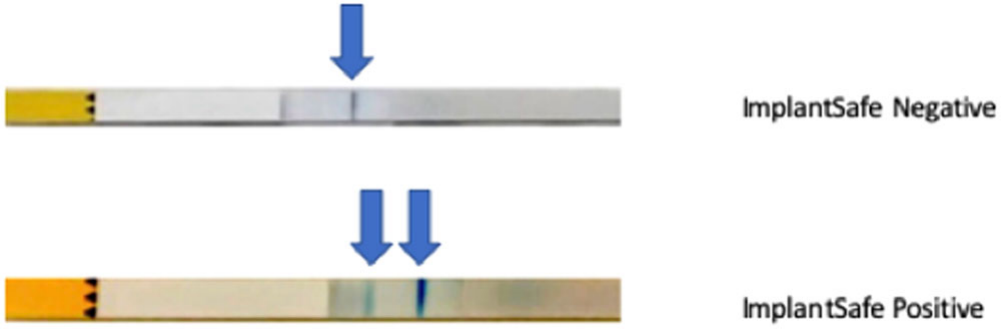
A site‐specific aMMP‐8 PoC lateral‐flow immunoassay gingival crevicular fluid (GCF) test. A negative (one blue line) test result indicates aMMP‐8 levels <20 ng/ml in GCF; and a positive test result (two blue lines) aMMP‐8 levels of ≥20 ng/ml in GCF. aMMP‐8, active matrix metalloproteinase 8; PoC, point‐of‐care.

The results in both cases were read as a single blue line indicating aMMP‐8 levels of less than 20 ng/ml (negative); and two blue lines as aMMP‐8 levels of more than 20 ng/ml (positive), indicating active periodontal disease (Figures 1 and 2).

### 2.5 Statistical analysis

Statistical analyses were performed using IBM SPSS Statistics for Windows, Version 27.0; IBM Corp., Armonk, NY. Results on continuous measurements were presented on mean ± SD (min–max) and categorical as frequency (percentage). The normality of the data was assessed using the Shapiro–Wilk test/Kolmogorov–Smirnov test. Bivariate associations were examined using Fisher's exact test/*χ* ^2^ test. Kruskal–Wallis test was used to compare the variables at a different level of periodontal disease. McNemar's test was used to compare the distribution of patients diagnosed as positive and negative based on kit results among the same patients who were tested for COVID‐19 initially and turned out negative later. Comparison was done with an intention to treat basis, that is it was done on those selective patients on whom before and after the kit was used. Criterion validity in terms of sensitivity, specificity, positive predictive value (PPV), and negative predictive value (NPV) with confidence intervals (CI 95%) were computed using a two‐way table with clinical diagnosis as a gold standard test. These diagnostics characteristics were calculated for aMMP‐8 PoC mouth rinse and site‐specific test kits with and without adjusting for the patient's age, gender, and smoking status. Adjusting for the three variables was calculated by logistic regression and the cut‐offs for predicted probabilities based on the Youden's index were further utilized in ROC analysis to assess the model's ability to classify patients' disease and health. The sensitivity of the test is its ability to identify correctly those patients who have the disease, whereas the specificity of the test is its ability to identify correctly those patients who do not have the disease. These two parameters are the most accepted ways to quantify the intrinsic diagnostic accuracy and validity of a medical test. However, in clinical practice, even if the sensitivity and specificity of a test are known, the clinician really wants to know how good the test is at predicting abnormality (i.e., what proportion of patients with an abnormal test result is truly abnormal). The PPV is the proportion of patients with a positive test result who truly have the disease and the NPV is the proportion of patients with negative test results who truly do not have the disease. However, PPVs and NPVs vary with the prevalence of a condition within a population while sensitivity and specificity are prevalence‐independent test characteristics. The significance level adopted was 5%.

## 3 RESULTS

Table 1, presents the association of various parameters with stages of periodontitis. A total of 72 COVID‐19 patients and 30 healthy patients were included in the present study. Age is significantly associated with stages of periodontitis in COVID‐19 patients while gender was not associated. Around 61.1% of patients had typical symptoms of COVID‐19 and 18.1% had ground glass opacities in computed tomography (CT) chest findings. COVID‐19 complications like hospital admission, assisted ventilation, and survival were seen more among patients with higher grades of periodontitis. Among the COVID‐19 negative cohort of 30 patients; age, gender, and presence of comorbidities were not significantly associated with stages of periodontitis in the present study. Thirty‐eight of the COVID‐19 positive patients, and six healthy subjects had comorbidities. In the 52.77% COVID‐19 positive patients with comorbidities, a statistically significant association was observed for diabetes mellitus, cardiovascular diseases, and cancer. Four of the seven deceased had one or more comorbidities. Three of the COVID‐19 positive patients were subjected to an antibiotic regimen in the last 3 months for the management of their infective systemic conditions.

**Table 1:** Patient characteristics.

	Parameters		Healthy (*n* = 27)	Gingivitis (*n* = 21)	Stage I (*n* = 3)	Stage I (*n* = 2)	Stage III (*n* = 14)	Stage IV (*n* = 5)	*p* Value
COVID‐19 positive cohort (*n* = 72)	Age (in years)	Mean ± SD	34.44 ± 11.06	37.71 ± 10.0	52.33 ± 16.25	44.00 ± 18.38	65.57 ± 12.32	65.40 ± 4.77	.001*
	Sex	Male *n* (%)	18 (25)	11 (15.3)	3 (4.2)	1 (1.4)	9 (12.5)	3 (4.2)	.732
		Female *n* (%)	9 (12.5)	10 (13.9)	0	1 (1.4)	5 (6.9)	2 (2.8)	
	Comorbidities	Present *n* (%)	12 (16.6)	8 (11.1)	2 (2.7)	2 (2.7)	11 (15.2)	3 (4.1)	.425
		Absent *n* (%)	15 (20.8)	13 (18.0)	1 (1.3)	0	3 (4.1)	2 (2.7)	
	COVID symptoms	Asymptomatic *n* (%)	9 (12.5)	5 (6.9)	1 (1.4)	2 (2.8)	8 (11.1)	3 (4.2)	.117
		Symptomatic *n* (%)	18 (25)	16 (22.2)	2 (2.8)	0	6 (8.3)	2 (2.8)	
	CT chest findings: Ground glass opacities	Present *n* (%)	3 (4.2)	3 (4.2)	1 (1.4)	0	5 (6.9)	1 (1.4)	.339
		Absent *n* (%)	24 (33.3)	18 (25)	2 (2.8)	2 (2.8)	9 (12.5)	4 (5.6)	
	Hospital admission	Home isolation *n* (%)	16 (22.2)	12 (16.7)	0	0	0	0	.001*
		Ward admission *n* (%)	11 (15.3)	6 (8.3)	3 (4.2)	2 (2.8)	7 (9.7)	4 (5.6)	
		ICU admission *n* (%)	0	3 (4.2)	0	0	7 (9.7)	1 (1.4)	
	Oxygen requirement	Room air *n* (%)	24 (33.3)	16 (22.2)	1 (1.4)	2 (2.8)	3 (4.2)	2 (2.8)	.001*
		HFNC/NIV *n* (%)	3 (4.2)	5 (6.9)	2 (2.8)	0	6 (8.3)	1 (1.4)	
		Ventilator/intubation *n* (%)	0	0	0	0	5 (6.9)	2 (2.8)	
	COVID pneumonia	Present *n* (%)	3 (4.2)	4 (5.6)	1 (1.4)	0	7 (9.7)	2 (2.8)	.071
		Absent *n* (%)	24 (33.3)	17 (23.6)	2 (2.8)	2 (2.8)	7 (9.7)	3 (4.2)	
	Survival	Survived *n* (%)	27 (37.5)	20 (27.8)	3 (4.2)	2 (2.8)	9 (12.5)	4 (5.6)	.010*
		Deceased *n* (%)	0	1 (1.4)	0	0	5 (6.9)	1 (1.4)	

	**Parameters**		**Healthy (*n*** = **7)**	**Gingivitis (*n*** = **10)**	**Stage I (*n*** = **0)**	**Stage II (*n*** = **3)**	**Stage III (*n*** = **4)**	**Stage IV (*n*** = **6)**	***p* Value**
COVID‐19 negative cohort (*n* = 30)	Age (in years)	Mean ± SD	38.29 ± 15.95	35.20 ± 8.36	0	34.33 ± 10.69	46.25 ± 9.39	50.83 ± 4.70	.052
	Sex	Male *n *(%)	2 (6.7)	4 (13.3)	0	2 (6.7)	1 (3.3)	1 (3.3)	.692
		Female *n *(%)	5 (16.7)	6 (20)	0	1 (3.3)	3 (10)	5 (16.7)	
	Comorbidities	Present *n* (%)	1 (3.3)	2 (6.7)	0	0	1 (3.3)	2 (6.7)	.890
		Absent *n *(%)	6 (20)	8 (26.7)	0	3 (10)	3 (10)	4 (13.3)	

*Note*: *p* Values calculated with Fisher's exact test/*χ* ^2^ test, and Kruskal–Wallis test.

Abbreviations: CT, computed tomography; HFNC, high flow nasal cannula; ICU, intensive care unit; NIV, noninvasive ventilation.

* Statistically significant (*p* < .05).

Table 2 presents the criterion validity of both the kits in the COVID‐19 positive and negative cohort. aMMP‐8 mouth rinse test had a sensitivity and specificity of 76.19% and 43.14%, respectively in the COVID‐19 positive cohort while the aMMP‐8 site‐specific test had a sensitivity and specificity of 61.90% and 58.82%. However, in the COVID‐19 negative cohort, for the aMMP‐8 mouth rinse test, the sensitivity and specificity were 84.62% and 17.65%, respectively, whereas for aMMP‐8 site‐specific test the sensitivity was 69.23% and specificity was 47.06%. Depending on the definition of diseased and healthy (Table 2), sensitivity increased as the severity of periodontitis increased, while specificity was highest for the healthiest. Furthermore, sensitivity increased among COVID‐19 negative patients when compared to COVID‐19 positive patients, while at the same time, specificity decreased.

**Table 2:** Criterion validity of aMMP‐8 mouth rinse test and aMMP‐8 site‐specific test kits.

		Test result	Stage II–IV	Healthy, gingivitis, Stage I	Sensitivity (%) (95% CI)	Specificity (%) (95% CI)	PPV (%) (95% CI)	NPV (%) (95% CI)
COVID‐19 positive cohort (*n* = 72)	aMMP‐8 mouthrinse test	Positive	16	29	76.19 (52.83–91.78)	43.14 (29.35–57.75)	35.56 (21.87–51.22)	81.48 (61.92–93.70)
		Negative	5	22				
	aMMP‐8 site‐specific test	Positive	13	21	61.90 (38.44–81.89)	58.82% (44.17%–72.42%)	38.24 (22.17–56.44)	78.95 (62.68–90.45)
		Negative	8	30				
COVID‐19 negative cohort (*n* = 30)	aMMP‐8 mouthrinse test	Positive	11	14	84.62 (54.55– 98.08)	17.65 (3.80– 43.43)	44.00 (24.40–65.07)	60.00 (14.66–94.73)
		Negative	2	3				
	aMMP‐8 site‐specific test	Positive	9	9	69.23 (38.57– 90.91)	47.06 (22.98–72.19)	50.00 (26.02–73.98)	66.67 (34.89–90.08)
		Negative	4	8				

		Test result	Stage I–IV	Healthy, gingivitis	Sensitivity (%) (95% CI)	Specificity (%) (95% CI)	PPV (%) (95% CI)	NPV (%) (95% CI)
COVID‐19 positive cohort (*n* = 72)	aMMP‐8 mouthrinse test	Positive	16	29	66.67 (44.68–84.37)	39.58 (25.77–54.73)	35.56 (21.87–51.22)	70.37 (49.82–86.25)
		Negative	8	19				
	aMMP‐8 site‐specific test	Positive	13	21	54.17 (32.82–74.45)	56.25 (41.18–70.52)	38.24 (22.17–56.44)	71.05 (54.10–84.58)
		Negative	11	27				
COVID‐19 negative cohort (*n* = 30)	aMMP‐8 mouthrinse test	Positive	11	14	84.62 (54.55–98.08)	17.65 (3.80–43.43)	44.00 (24.40–65.07)	60.00 (14.66–94.73)
		Negative	2	3				
	aMMP‐8 site‐specific test	Positive	9	9	69.23 (38.57–90.91)	47.06 (22.98–72.19)	50.00 (26.02–73.98)	66.67 (34.89–90.08)
		Negative	4	8				

		Test result	Stage I–IV	Healthy, gingivitis	Sensitivity (%) (95% CI)	Specificity (%) (95% CI)	PPV (%) (95% CI)	NPV (%) (95% CI)
COVID‐19 positive cohort (*n* = 72)	aMMP‐8 mouthrinse test	Positive	29	16	64.44 (48.78–78.13)	40.74 (22.39–61.20)	64.44 (48.78–78.13)	40.74 (22.39–61.20)
		Negative	16	11				
	aMMP‐8 site‐specific test	Positive	24	10	53.33 (37.87–68.34)	62.96 (42.37–80.60)	70.59 (52.52–84.90)	44.74 (28.62–61.70)
		Negative	21	17				
COVID‐19 negative cohort (*n* = 30)	aMMP‐8 mouthrinse test	Positive	20	5	86.96 (66.41–97.22)	28.57 (3.67–70.96)	80.00 (59.30– 93.17)	40.00 (5.27–85.34)
		Negative	3	2				
	aMMP‐8 site‐specific test	Positive	14	4	60.87 (38.54–80.29)	42.86 (9.90–81.59)	77.78 (52.36–93.59)	25.00 (5.49–57.19)
		Negative	9	3				

Abbreviations: aMMP‐8, active matrix metalloproteinase 8; CI, confidence interval; NPV, negative predictive value; PPV, positive predictive value.

**Figure 3:**
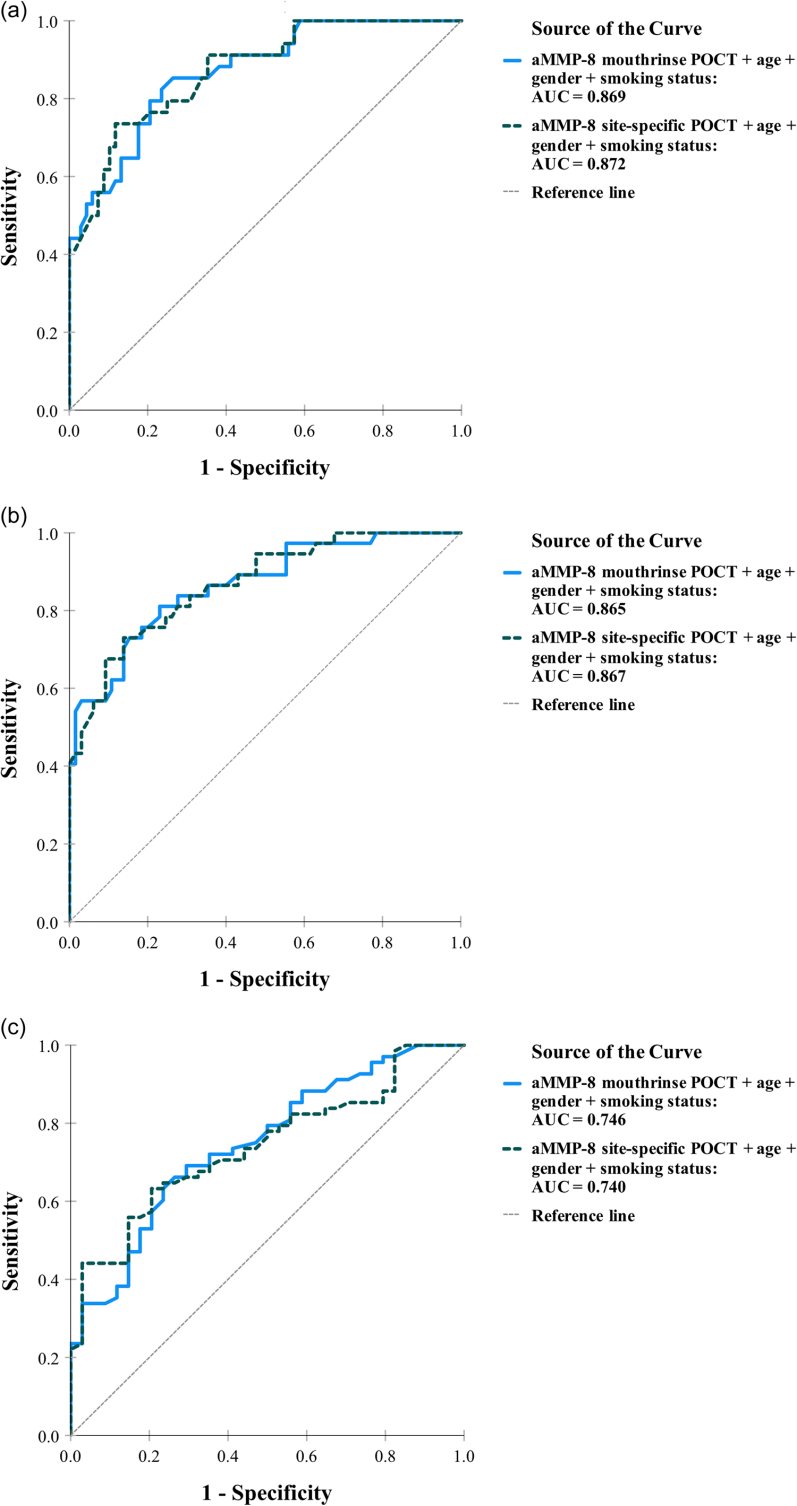
Receiver operating characteristic (ROC) curves and the area under the ROC curve (AUC) calculated for the logistic regression models comprising the qualitative aMMP‐8 PoC mouth rinse and site‐specific test kits (cut‐off of 20 ng/ml) adjusted for periodontal disease risk factors age, gender and smoking status (*N* = 102). The ROC analysis represents the ability to classify patients into (a) healthy + gingivitis + Stage I periodontitis versus Stage II–IV periodontitis, (b) healthy + gingivitis versus Stage I–IV periodontitis, and (c) healthy versus gingivitis + Stage I–IV periodontitis. aMMP‐8, active matrix metalloproteinase 8; PoCT, point‐of‐care test.

Table 3 shows the overall criterion validity among 102 patients for both kits. Sensitivity was higher for mouth rinse kit (79.41% > 64.71%) while specificity increased in site‐specific kit (55.88% > 36.76%). When adjusted for age, gender, and smoking status both sensitivity and specificity of both aMMP‐8 kits increased. The AUCs from the ROC analysis ranged between 0.746–0.869 (*p* < .001) and 0.740–0.872 (*p* < .001) for the aMMP‐8 mouth rinse and site‐specific kits, respectively, adjusted for age, gender, and smoking status (Figure 3). Similar to Table 2, depending on the definition of diseased and healthy, the AUCs, as well as the overall sensitivity, increased as the severity of periodontitis increased, while specificity was highest for the healthiest patients.

**Table 3:** Overall criterion validity of aMMP‐8 mouthrinse test and aMMP‐8 site‐specific test kits with and without adjusting for age, gender, and smoking status (*N* = 102).

	Test result	Stage II–IV	Healthy, gingivitis, Stage I	Sensitivity (%) (95% CI)	Specificity (%) (95% CI)	PPV (%) (95% CI)	NPV (%) (95% CI)
aMMP‐8 mouthrinse test	Positive	27	43	79.41 (62.10–91.30)	36.76 (25.39–49.33)	38.57 (27.17–50.97)	78.13 (60.03–90.72)
	Negative	7	25				
aMMP‐8 mouthrinse test (age, gender, and smoking adjusted)^a^	Positive	28	16	82.35 (65.47–93.24)	76.47 (64.62–85.91)	63.64 (52.59–73.41)	89.66 (80.56–94.77)
	Negative	6	52				
aMMP‐8 site‐specific test	Positive	22	30	64.71 (46.49–80.25)	55.88 (48.64–68.48)	42.31 (28.73–56.80)	76.00 (61.83–86.94)
	Negative	12	38				
aMMP‐8 site‐specific test (age, gender, and smoking adjusted)^a^	Positive	25	8	73.53 (55.64–87.12)	88.24 (78.13–94.78)	75.76 (61.25–86.07)	86.96 (79.09–92.16)
	Negative	9	60				

	Test result	Stage I–IV	Healthy, gingivitis	Sensitivity (%) (95% CI)	Specificity (%) (95% CI)	PPV (%) (95% CI)	NPV (%) (95% CI)
aMMP‐8 mouthrinse test	Positive	27	43	72.97 (55.88–86.21)	33.85 (22.57–46.65)	38.57 (27.17–50.97)	68.75 (49.99–83.88)
	Negative	10	22				
aMMP‐8 mouthrinse test (age, gender, and smoking adjusted)^a^	Positive	30	15	81.08 (64.84–92.04)	76.92 (64.81–86.47)	66.67 (55.55–76.20)	87.72 (78.35–93.38)
	Negative	7	50				
aMMP‐8 site‐specific test	Positive	22	30	59.46 (42.10–75.25)	53.85 (41.03–66.30)	42.31 (28.73–56.80)	70.00 (55.39–82.14)
	Negative	15	35				
aMMP‐8 site‐specific test (age, gender, and smoking adjusted)^a^	Positive	27	9	72.97 (55.88–86.21)	86.15 (75.34–93.47)	75.00 (61.33–85.02)	84.85 (76.57–90.56)
	Negative	10	56				

	Test result	Stage I–IV	Healthy, gingivitis	Sensitivity (%) (95% CI)	Specificity (%) (95% CI)	PPV (%) (95% CI)	NPV (%) (95% CI)
aMMP‐8 mouthrinse test	Positive	49	21	72.06 (59.85–82.27)	38.24 (22.17–56.44)	70.00 (57.87–80.38)	40.63 (23.70–59.36)
	Negative	19	13				
aMMP‐8 mouthrinse test (age, gender, and smoking adjusted)^a^	Positive	45	9	66.18 (53.68–77.21)	73.53 (55.64–87.12)	83.33 (73.58–89.98)	52.08 (42.42–61.59)
	Negative	23	25				
aMMP‐8 site‐specific test	Positive	38	14	55.88 (43.32–67.92)	58.82 (40.70–75.35)	73.08 (58.98–84.43)	40.00 (26.41–54.82)
	Negative	30	20				
aMMP‐8 mouthrinse test (age, gender, and smoking adjusted)^a^	Positive	43	7	63.24 (50.67–74.61)	79.41 (62.10–91.30)	86.00 (75.60–92.41)	51.92 (43.08–60.65)
	Negative	25	27				

Abbreviations: aMMP‐8, active matrix metalloproteinase 8; CI, confidence interval; NPV, negative predictive value; PPV, positive predictive value.

^a^ Age, gender, and smoking status adjusted logistic regression model, the optimal cut‐off for probabilities by Youden's index.

Table 4 compared aMMP‐8 mouth rinse and site‐specific test kits and found a significant association between the two kits (odds ratio = 5.077, *p* < .001). A mouth rinse test was more likely to indicate the risk of active collagenolysis for a patient (a positive test result) compared with a site‐specific test (*p* = .003).

**Table 4:** The association between aMMP‐8 mouthrinse test and aMMP‐8 site‐specific test kits (*N* = 102).

		aMMP‐8 site‐specific test							
	Test result	Positive	Negative	Odds ratio (95% CI)	*p* Value	Sensitivity (%) (95% CI)	Specificity (%) (95% CI)	PPV (%) (95% CI)	NPV (%) (95% CI)
aMMP‐8 mouthrinse test	Positive	44	26	5.077 (1.992–12.939)	0.003	84.62 (71.92–93.12)	48.00 (33.66–62.58)	62.86 (50.48–74.11)	75.00 (56.60–88.54)
	Negative	8	24						

*Note*: *p* Values calculated by McNemar's test.

Abbreviations: aMMP‐8, active matrix metalloproteinase‐8; CI, confidence interval; NPV, negative predictive value; PPV, positive predictive value.

**Figure 4:**
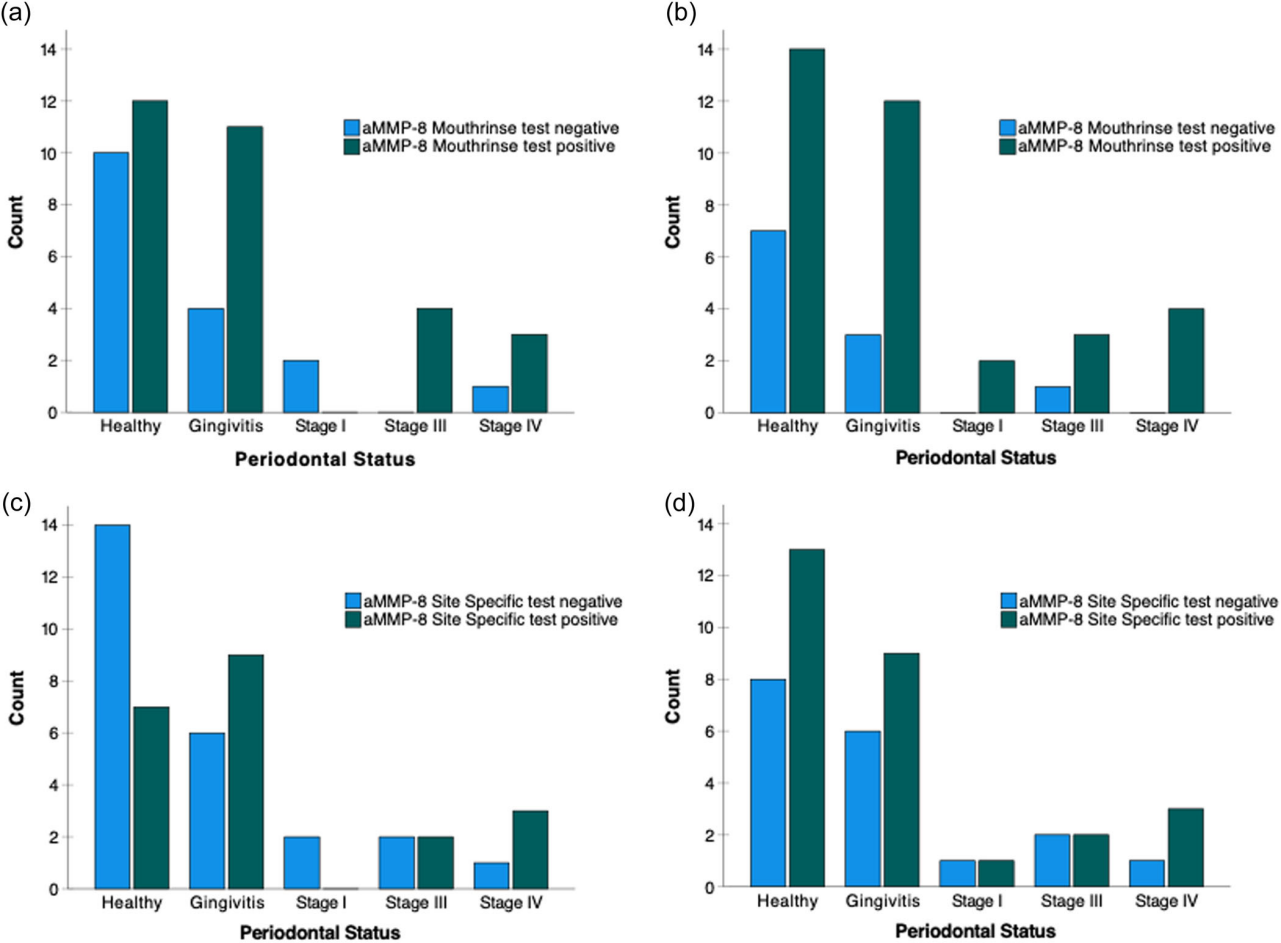
Mouthrinse aMMP‐8 PoC test results categorized by periodontal status (a) of COVID‐19 positive patients and (b) after their recovery (a negative COVID‐19 test). Site‐specific aMMP‐8 PoC test results categorized by periodontal status (c) of COVID‐19 positive patients and (d) after their recovery (a negative COVID‐19 test). aMMP‐8, active matrix metalloproteinase 8; PoCT, point‐of‐care.

Figure 4 and Table 5 depict the aMMP‐8 mouth rinse and site‐specific test results before and after a COVID‐19 negative test result in the COVID‐19 cohort. A positive aMMP‐8 mouth rinse and site‐specific test results were more frequent regardless of the prior aMMP‐8 test result when checked once patients had recovered from COVID‐19. However, the difference in the distribution of aMMP‐8 test results for both kits did not reach statistical significance (*p* = .302 and *p* = .189, respectively).

**Table 5:** COVID‐19 positive patients were tested by aMMP‐8 PoC/chairside (site‐specific gingival crevicular fluid (GCF) and whole mouth specific mouth rinse) tests when patients were COVID‐19 positives (RT‐PCR+) and after their recovery to COVID‐19 negatives (RT‐PCR−) and the association between the aMMP‐8 test result before and after COVID‐19 (related samples) were compared.

	aMMP‐8 mouthrinse test result	Negative	Positive	*p* Value	Odds ratio (95% CI)
		RT‐PCR−			
RT‐PCR+	Negative (*n* = 16)	6	10	.302	3.000 (0.743–12.106)
	Positive (*n* = 30)	5	25		
		RT‐PCR−			
RT‐PCR+	Negative (*n* = 25)	11	14	.189	1.571 (0.472–5.232)
	Positive (*n* = 21)	7	14		

*Note*: *p* Values calculated by McNemar's test.

Abbreviations: aMMP‐8, active matrix metalloproteinase 8; CI, confidence interval; RT‐PCR, reverse transcription‐polymerase chain reaction.

## 4 DISCUSSION

The findings of the present study seem to be quite encouraging as far as the establishment of an objective PoC screening diagnostic methodology is concerned. The mouth rinse and site‐specific GCF‐based kits were able to detect periodontal disease with moderate to high sensitivity and specificity depending on the definition of periodontally diseased and healthy. The sensitivity increased when the diseased were defined as severe stages of periodontitis, while specificity peaked in the healthiest patients. The aMMP‐8 mouth rinse test had higher sensitivity but lower specificity compared with the site‐specific aMMP‐8 test in the COVID‐19 positive and negative groups as well as overall. When the aMMP‐8 PoC test results of mouth rinse and site‐specific kits were adjusted for the three risk factors for periodontitis, the age, gender, and smoking status of patient, precision for periodontitis was even better and sensitivity and specificity were higher ranging between 63.24%–82.35% and 73.53%–88.24%, respectively, depending on the definition of the periodontal disease and health.

The sensitivity of the kits in the COVID negative cohort is similar to that reported in the literature (Sorsa et al., 2015, 2017). The comparative decrease in the specificity of the kits to measure active periodontal disease in the COVID‐19 positive cohort could be due to the additional elevation in the aMMP‐8 levels due to the ensuing viral‐induced cytokine storm in COVID‐19 patients. Compromised oral hygiene during COVID‐19 may play a role here, as well. Hence, the relatively small PPV of the mouth rinse and site‐specific aMMP‐8 kits run in the COVID‐19 positive cohort, in fact, indicates that few of the positive results from this testing procedure are false positives for the same reason. COVID‐19 and poor oral hygiene alone or together may induce gingival and/or periodontal inflammation simultaneously activating collagenolysis in the periodontium that was detected by elevated aMMP‐8 levels. In this regard, the number of positive aMMP‐8 test results of mouth rinse and site‐specific tests increased markedly once the virus became undetectable at RT‐PCR compared with the initial aMMP‐8 test results. This suggests an increased susceptibility to active periodontal breakdown and risk of progression of attachment loss because of COVID‐19 and/or compromised oral hygiene during COVID‐19. However, possibly because of the relatively small sample size, the difference was not significant here but nevertheless was observable (Figure 4). Here, a relatively small sample size is a limitation in this study which resulted in decreased post hoc power. Thus, further studies are required in larger populations.

The strength of this screening test is also in its high NPV (78.13% for the mouth rinse test and 76.00% for the site‐specific test) which, if negative for an individual, gives us high confidence that its negative result is true. The high NPV of the kits, hence justifies its mandate as a screening test for periodontal disease in COVID‐19 patients, to be confirmed with a more detailed clinical/radiological examination in those testing positive. Furthermore, adjusting the aMMP‐8 mouth rinse and site‐specific test results for risk factors of periodontitis that is patient's age, gender, and smoking status were able to increase markedly both PPV and NPV of the kits to detect more accurately patients with clinical signs of periodontal disease. It should be noted that both aMMP‐8 test kits are designed to identify patients with active collagenolysis and periodontal breakdown at site‐specific and full‐mouth levels. As such, a positive aMMP‐8 test result may be a signal of initiating or progressing periodontal disease. Previous prospective studies have shown the importance of aMMP‐8 in the progression of periodontal disease and attachment loss (Gupta, Sahni et al., 2021; Keskin et al., 2020; Lee et al., 1995; Leppilahti et al., 2014, 2015; Mancini et al., 1999; Räisänen et al., 2021; Romanelli et al., 1999; Sorsa et al., 2020).

MMPs have been held responsible for bringing about the degradation of the extracellular matrix as well as remodeling of pulmonary tissue. This generally promotes vascular permeability and damage to the endothelium at the level of the basal lamina. To further complicate matters, the management of ARDS involves assisted ventilation which can itself lead to pulmonary injury mediated via the MMP pathway. Not only this, but MMP‐8 levels have also been evidenced to be increased alongside MMP‐9, MMP‐2, and TIMP‐1 during early sepsis, a condition which has eventually been related to patient mortality (Malek et al., 2020).

The MMP pathway of inflammation is not only at play during acute presentations but also emerges and assumes importance in a more chronic and low‐grade variety such as that involving obesity. Metabolic syndrome and diabetes (often a result of the former) have been associated with COVID‐19‐related adverse outcomes, and both these conditions have been linked with elevated MMP‐8 levels. MMP‐8 brings about human insulin receptor protein cleavage which in turn results in insulin resistance and consequently, diabetes mellitus. There is thus, ample evidence in the literature to justify further studies regarding the potential involvement of MMP‐8 in the pathophysiology of COVID‐19 infections (Sahni & Gupta 2020; Räisänen et al., 2020).

The present study found that age was significantly associated with the severity of periodontitis in COVID‐19 patients, a fact well founded both in logic and literature (Centers for Disease Control and Prevention [CDC], 2021). The study, however, found no association between the severity of periodontitis and COVID‐19 in terms of gender. This differs from literature which predilects the severity of infection to the male gender (Griffith et al., 2020). Moreover, the proportion of smokers was relatively low in this sample. Nevertheless, taking into account these three risk factors of periodontitis age, gender, and smoking status together with the aMMP‐8 POCT was able to increase the accuracy of identifying periodontal disease and health. This is in agreement with and further extends previous studies demonstrating the benefits of assessing aMMP‐8 POCT measurements together with the other risk factors of periodontitis when screening for periodontitis and related history of occurred attachment loss (Räisänen et al., 2021).

COVID‐19‐related adverse outcomes seemed to have an association with the severity of periodontitis, this is in agreement with a few previous studies suggesting a similar relationship (Gupta, Saarikko et al., 2022; Gupta, Sorsa et al., 2022; Marouf et al., 2021). Chest findings on CT scans are fast emerging as a standardized methodology to diagnose COVID‐19 infections in patients who have tested negative on RT‐PCR but otherwise remain symptomatic in favor of COVID‐19 (Pakdemirli et al., 2020). In our study, the majority of patients were symptomatic with a significant number presenting with ground‐glass opacities as CT‐chest findings. Demographic and comorbidity‐related factors, however, remained unassociated with the severity of periodontitis in patients who were deemed to be COVID‐19 negative. This finding also differs from a number of established reports in the literature but can be attributed to the small sample size.

Seeing as the presence of periodontitis seems to correlate with a worsening of COVID‐19 related adverse outcomes, the utilization of the aMMP‐8 PoC diagnostic kits, assumes importance as a screening tool to diagnose active periodontal disease states at both the full‐mouth and site‐specific levels. As Sampson et al. (2020) discuss, a raised level of association between severe COVID‐19 infection and the presence of chronic periodontitis might be accounted for by the phenomenon first identified by Limeback, (1988a, 1998b)—there is greater potential for aspiration of periodontitis‐associated organisms directly into the lungs if subjects have chronic periodontitis (Scannapieco & Genco, 1999).

There was a significant association between mouth rinse and site‐specific aMMP‐8 tests. A mouth rinse test was also more likely to give a positive test result compared with a site‐specific test. The results indicate that the mouth rinse aMMP‐8 test can detect the site with maximum periodontal breakdown with enough precision. Thus, full‐mouth disease activity measured by the mouth rinse test can benefit the identification of undiagnosed patients with potentially compromised oral health. Also, the PoC methodology would find use in the hands of nondental medical professionals to stratify risk groups, decide on referrals and contemplate the institution of oral hygiene in their patients as a dental or specialist periodontal referral may not be possible in every situation. The whole process using aMMP‐8 PoC testing with or without simple questionnaire(s) can be regarded as wholly noninvasive without risk of bacteremia taking only 5–10 min of time. There is evidence in the literature of a validated questionnaire and PoC aMMP‐8 based diagnostic toolkit for medical practitioners (Räisänen et al., 2021). The results in the present study with a similar noninvasive screening strategy are very promising considering that the quantitative aMMP‐8 POCT measurements were not available in this study. Using qualitative results instead of quantitative measurements eventually decreased the power to find significant differences as well as decreased the diagnostic accuracy. Regardless, as our results show, such a noninvasive screening methodology can prove to be invaluable in times of a pandemic wherein the simplicity of its conduction and output may enable any medical or paramedical professional as well as laymen to diagnose the presence of active disease and take appropriate action.

## 5 CONCLUSIONS

The findings of the present study recommend the utilization of an aMMP‐8‐based PoC diagnostic methodology as a screening tool to ascertain the presence of active periodontal disease in COVID‐19 patients as well as individuals spared from the pandemic. The tool is simplistic and objective to enable its use by medical and paramedical professionals as well as laymen. Combination with information about patients' risk factors of periodontitis may increase the accuracy of this screening strategy even further. The noninvasive identification of periodontal disease in this manner would aid in stratifying risk groups, deciding referrals, and in devising oral hygiene maintenance recommendations during the COVID‐19 pandemic.

## AUTHOR CONTRIBUTIONS

Shipra Gupta, contributed to conception, design, data acquisition, analysis, and interpretation, drafted the manuscript and critically revised the manuscript. Ritin Mohindra contributed to acquisition and critically revised the manuscript. Mini P. Singh, contributed to acquisition and drafted the manuscript. Sagar Khera contributed to acquisition and critically revised the manuscript. Amit Kumar contributed to interpretation and drafted the manuscript. Nilminie Rathnayake contributed to interpretation and critically revised the manuscript. Timo Sorsa contributed to interpretation and critically revised the manuscript. Andreas Pfützner contributed to interpretation and critically revised the manuscript. Ismo T. Räisänen contributed to interpretation and critically revised the manuscript. Roop K. Soni contributed to acquisition and critically revised the manuscript. Poonam Kanta contributed to analysis and critically revised the manuscript. Akanksha Jain contributed to analysis and critically revised the manuscript. Krishan Gauba contributed to interpretation and critically revised the manuscript. Kapil Goyal contributed to analysis and critically revised the manuscript. Mini P. Singh contributed to analysis and critically revised the manuscript. Arnab Ghosh contributed to acquisition and critically revised the manuscript. Kamal Kajal contributed to acquisition and critically revised the manuscript. Varun Mahajan contributed to acquisition and critically revised the manuscript. Vikas Suri contributed to interpretation and critically revised the manuscript. Ashish Bhalla contributed to interpretation and critically revised the manuscript. All authors gave final approval and agreed to be accountable for all aspects of the work. All authors have read and agreed to the published version of the manuscript.

## CONFLICTS OF INTEREST

Timo Sorsa is the inventor of US patents 5652223, 5736341, 5864632, 6143476, 2017/0023571A1 (issued June 6, 2019), WO2018/060553A1 (issued May 31, 2018), 10488415B2, and a Japanese patent 2016‐554676. The other authors report no conflict of interest with respect to the research, authorship, and/or publication of this article. The funders had no role in the design of the study; in the collection, analyses, or interpretation of data; in the writing of the manuscript, or in the decision to publish the results.

## ETHICS STATEMENT

The study was conducted according to the guidelines of the Declaration of Helsinki, and approved by the Institute Ethics Committee of Postgraduate Institute of Medical Education and Research (PGIMER), Chandigarh, India (INT/IEC/2021/SPL‐453 & 636). Informed consent was obtained from all subjects involved in the study.

## Data Availability

The data that support the findings of this study are available on reasonable request from the corresponding author. The data are not publicly available due to privacy and ethical restrictions.
